# Asthma Control Among Adults in Saudi Arabia: A Systematic Review and Meta-Analysis

**DOI:** 10.3390/jcm14165753

**Published:** 2025-08-14

**Authors:** Mohammed M. Alqahtani, Mansour M. Alotaibi, Saeed Mardy Alghamdi, Ali Alammari, Jameel Hakeem, Fawzeah Alenazi, Nour Aldhaefi, Deema Faleh Almutairi, Ahad Adel Alghamdi, Hamdan Al-Jahdali

**Affiliations:** 1 Department of Respiratory Therapy, College of Applied Medical Sciences, King Saud bin Abdulaziz University for Health Sciences, Riyadh 22384, Saudi Arabia; alenazifauzeah@gmail.com (F.A.); nouraldhaefi@gmail.com (N.A.); deema.f@outlook.sa (D.F.A.); ahad.adel2002@gmail.com (A.A.A.); 2 King Abdullah International Medical Research Center, Riyadh 22384, Saudi Arabia; jahdalih@ngha.med.sa; 3 Department of Respiratory Services, King Abdulaziz Medical City, Ministry of National Guard Health Affairs, Riyadh 22384, Saudi Arabia; 4 Department of Rehabilitation, Faculty of Applied Medical Sciences, Northern Border University, Arar 91431, Saudi Arabia; mansour.alotaibi@nbu.edu.sa; 5 Center for Health Research, Northern Border University, Arar 91431, Saudi Arabia; 6 Clinical Technology Department, Respiratory Care Program, Faculty of Applied Medical Sciences, Umm Al-Qura University, Makkah 24382, Saudi Arabia; smghamdi@uqu.edu.sa; 7 Department of Rehabilitation Sciences, University of Alabama at Birmingham, Birmingham, AL 35294, USA; alammari@uab.edu; 8 Department of Respiratory Care, King Fahd Hospital, Jeddah 23325, Saudi Arabia; 9 Department of Respiratory Therapy, College of Applied Medical Sciences, King Saud bin Abdulaziz University for Health Sciences, Jeddah 22384, Saudi Arabia; hakeemj@ksau-hs.edu.sa; 10 King Abdullah International Medical Research Center, Jeddah 22384, Saudi Arabia; 11 Department of Medicine, Pulmonary Division, King Abdulaziz Medical City, King Saud Bin Abdulaziz University for Health Sciences, Riyadh 22384, Saudi Arabia

**Keywords:** asthma control, adults, Saudi Arabia, systematic review, uncontrolled, partially controlled, controlled asthma

## Abstract

**Background/Objectives:** Asthma is a condition caused by chronic lower airway inflammation. Its primary treatment focuses on managing the condition and reducing the frequency of exacerbation episodes. Monitoring the level of asthma control among adults is essential for both clinical care and public health planning. This systematic review aimed to assess the level of asthma control among adults in Saudi Arabia and to determine the prevalence of controlled asthma in this population. **Methods:** The literature search was conducted using PubMed. We included all English-language, empirical, quantitative studies that investigated the prevalence of asthma control among Saudi adults. National Institutes of Health (NIH) Study Quality Assessment Tools guided determination of the quality of the included studies. This review is registered with PROSPERO (CRD42024484711). **Results:** Of the 107 initially identified studies, 17 met the inclusion criteria. Quality assessment tool rated 11 studies as good, 5 as fair, and 1 as poor. Most of the included studies used cross-sectional design from different geographical locations and varied in sample size. Overall, the prevalence of uncontrolled asthma among Saudi adults ranged from 23.4% to 68.1%. In some studies, well-controlled asthma was reported in as few as 3% of patients. Factors associated with uncontrolled asthma included lower educational attainment, unemployment, low income, female gender, tobacco use, poor medication adherence, and lack of regular medical follow-up. Environmental triggers and comorbid conditions, such as allergic rhinitis, were also frequently cited as contributing factors. **Conclusions:** Asthma control among adults in Saudi Arabia remains a significant public health concern. Improving outcomes requires a multifaceted approach that includes patient education, regular follow-up care (including pulmonary function tests, asthma severity assessments, and personalized treatment plans), and broader public health initiatives aimed at reducing exposure to allergens and pollutants. Strengthening primary care services and implementing nationwide asthma management programs may play a critical role in enhancing disease control and improving quality of life. Continued research in this field is strongly recommended.

## 1. Background

Asthma is a chronic respiratory condition characterized by inflammation of the lower airways, resulting in symptoms such as shortness of breath, wheezing, coughing, and airway obstruction [1,2]. While it was traditionally considered a single disease with a standardized treatment approach, asthma is now recognized as a complex and heterogeneous disorder with various phenotypes and underlying mechanisms [2]. The Global Initiative for Asthma (GINA) has identified distinct phenotypes based on demographic, clinical, and pathophysiological characteristics. These include allergic asthma, non-allergic asthma, late-onset asthma, asthma with fixed airflow limitation, and asthma associated with obesity [2]. Identifying these phenotypes is critical for guiding targeted therapies and achieving better asthma control. However, there is still limited understanding of how these phenotypes relate to disease progression and treatment response [2]. Various factors contribute to the development and exacerbation of asthma, including respiratory infections, cold air, allergens, genetic predispositions, obesity, and exposure to tobacco smoke [2]. The immune response—mediated by multiple cell types such as macrophages, mast cells, eosinophils, neutrophils, T lymphocytes, and epithelial cells—plays a central role in the airway inflammation characteristic of asthma [3]. Asthma can be broadly classified into allergic and non-allergic subtypes, with the presence of IgE antibodies typically indicating allergic asthma [3]. The prevalence of asthma varies widely across countries due to environmental exposures, as well as differences in measurement tools and epidemiological definitions. Several studies have assessed the prevalence of asthma and asthma-related symptoms among adults in Saudi Arabia. For example, Al Ghobain et al. conducted a study in Riyadh using the European Community Respiratory Health Survey (ECRHS) questionnaire to evaluate asthma prevalence [4]. The study found that 18.2% of participants reported wheezing or chest whistling in the past 12 months, with no significant gender differences. Asthma diagnosed by a physician was reported by 11.3% of participants, and 10.6% were actively taking asthma treatment at the time of the survey. The study also reported a 33.3% prevalence of nasal allergic reactions. Common asthma symptoms included morning chest tightness (33%), dyspnea (31%), coughing fits (43%), and recent asthma episodes (5.6%) [4]. Another study by Moradi Lakeh et al. aimed to estimate the national prevalence of asthma in Saudi Arabia using a random sampling technique [5]. The study found an estimated asthma prevalence of 4.05% among individuals aged 15 years and older, highlighting a substantial number of Saudis living with the condition. Uncontrolled asthma significantly impairs patients’ quality of life and underscores the need for effective management and strategies to prevent exacerbations [6]. Providing patients with adequate education and guidance on asthma self-management can empower them to manage mild symptoms at home and reduce the frequency of daytime asthma attacks [7]. However, studies have shown that a significant proportion of individuals in Saudi Arabia have uncontrolled asthma, with only a small percentage successfully managing their condition [6]. This alarming discrepancy highlights the urgent need for improved asthma management and control strategies across the country. Limited research and small sample sizes have contributed to the lack of comprehensive data on asthma control in the Middle East, specifically in Saudi Arabia [8]. Asthma affects not only physical health but also broader aspects of daily life, leading to missed school and workdays, hospitalizations, emergency department visits, and increased financial burden [2]. Moreover, individuals with asthma often face ongoing challenges, including morning symptoms, muscle soreness, and persistent anxiety [9,10,11].

This study aims to provide a comprehensive overview of the prevalence of controlled asthma among adults in Saudi Arabia. By examining the impact of asthma on daily activities and health-related outcomes, this study provides a valuable reference for researchers, respiratory therapists, pulmonologists, and other healthcare providers engaged in asthma care and health policy development.

## 2. Methods

### 2.1. Study Design

This systematic review was carried out following the guidelines of the Preferred Reporting Items for Systematic Reviews and Meta-Analyses (PRISMA). This review examined the prevalence of asthma control among adults in Saudi Arabia. The study protocol was registered with PROSPERO (registration number: CRD42024484711) (see Supplementary Materials, Figure S1, for more details).

### 2.2. Data Source

The literature search was conducted using PubMed (https://pubmed.ncbi.nlm.nih.gov, accessed on 15 March 2024). We included all English-language, empirical quantitative studies that investigated the prevalence of asthma control among Saudi adults. The quality of the identified studies was assessed using the NIH Study Quality Assessment Tools (https://www.nhlbi.nih.gov/health-topics/study-quality-assessment-tools, accessed on 15 March 2024).

### 2.3. Study Selection

Data were collected from relevant databases by four reviewers (D.A., A.A., F.A., and N.A.) using PubMed. Each reviewer independently screened all titles and abstracts based on the predefined inclusion and exclusion criteria. Full-text articles were then reviewed, and any disagreements were resolved by a fifth reviewer (M.A.).

### 2.4. Inclusion Criteria

In this systematic review, we included observational or intervention studies in which the study population consisted of adults aged 18 years and older with asthma. Asthma diagnosis in the included studies was based either on self-reported responses—where participants were asked whether they had ever been diagnosed with asthma—or on a confirmed clinical diagnosis by a physician. Additionally, studies that reported asthma-related respiratory symptoms such as breathlessness, dyspnea, breathing difficulties, wheezing, coughing, sputum production, and phlegm were also included (see Table 1 for more details).

### 2.5. Exclusion Criteria

**Figure 1:**
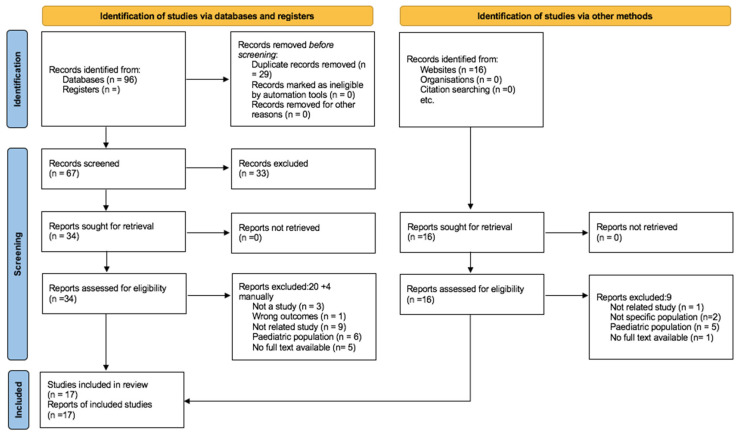
PRISMA flowchart for searches of databases, registers, and other sources.

This systematic review excluded publications that involved animal studies or did not report on adult asthma patients or asthma-related respiratory symptoms. Furthermore, any published systematic reviews (but screened the reference lists), non-English, manuscripts, non-Saudi Arabia research, conference abstracts with no full-text and non-full text articles were also excluded from the study (see Figure 1 for more details).

### 2.6. Data Extraction and Quality Assessment

In each included study, data extraction for all relevant outcomes was conducted independently by four reviewers (XX, YY, XY, and YX). These reviewers also gathered details on study characteristics, including the research design (e.g., interventional, cross-sectional, observational, or experimental), characteristics of the study population, participant age, and methods used to assess asthma control. Risk of bias (ROB) was evaluated independently by three reviewers (XX, YY, and XY) at both the study and outcome levels using the NIH Study Quality Assessment Tools. Studies were classified as having low risk of bias if they provided a thorough assessment and implemented appropriate adjustments for study-related factors (refer to Supplementary Materials, Table S1, for additional details).

## 3. Results

### 3.1. Quality Assessment and Risk of Bias

The included studies employed a variety of designs, including cross-sectional and randomized controlled trials, and incorporated both qualitative and quantitative research methodologies (see Table 2). Each study was evaluated using standardized quality assessment tools, specifically the NIH Quality Assessment Tool. Based on these assessments, 14 studies were rated as fair, 8 as good, and 2 as poor (see Supplementary Materials, Table S1, for more details).

### 3.2. Study Selection and Characteristics

Out of 94 eligible studies, only 17 met the inclusion criteria. Of these, nine were conducted in Riyadh, one in the Aseer Region, one in Jeddah, one in Najran, one in Al-Baha, and two in other Middle Eastern countries. Additionally, one study was conducted across both Jazan and Jeddah.

Among the 17 studies reviewed for the systematic review, 2 studies had sample sizes of fewer than 100 participants, 11 studies had sample sizes ranging from 100 to just under 1000 participants, and 4 studies involved sample sizes exceeding 1000 participants. The smallest study sample size was 53 [23], and it was 7955 in the largest study [17] (Figure 1).

### 3.3. Prevalence of Asthma Control

Among the 17 included studies, asthma control was most commonly assessed using the Arabic version of the Asthma Control Test (ACT) (n = 10 [6,12,14,15,16,19,21,22,23,26]), followed by the Global Initiative for Asthma (GINA) guidelines (n = 3 [8,24,25]), Mini Asthma Quality of Life Questionnaire (MiniAQLQ) (n = 1 [20]), objective clinical measures such as FeNO and spirometry in conjunction with ACT (n = 1 [23]), and modified ISAAC or Global Asthma Network (GAN)-based symptom questionnaires (n = 3 [13,17,18]). Asthma control among Saudi adults was found to be suboptimal in most of the studies. The prevalence of uncontrolled asthma was reported in six studies and ranged from 23.4% to 68.1% [6,12,14,15,25,27]. The prevalence of asthma control varied significantly across different studies conducted in Saudi Arabia. According to Ahmed A.E. (2014), the average asthma control score was 17.5 (±3.8), with control levels influenced by factors such as the use of inhaled corticosteroids (ICSs), consistency in follow-up, and education about asthma [12]. Alanazi et al. (2021) reported that among 200 asthma patients, 33.5% had well-controlled asthma, 27.5% had partially controlled asthma, and 39% had uncontrolled asthma [27]. In a study by Al-Jahdali et al. (2008) involving 1060 participants, 64% had uncontrolled asthma, 31% had well-controlled asthma, and 5% had completely controlled asthma [14]. Another study by Al-Jahdali et al. (2012) found that 23.4% of participants had uncontrolled asthma, 74.4% had partially controlled asthma, and 1.8% had completely controlled asthma [15]. BinSaeed (2015) observed that 68.1% of 260 patients had uncontrolled asthma [6]. Similarly, Tayeb et al. (2017) reported that 63% of their study population had uncontrolled asthma, 34% had partially controlled asthma, and only 3% had well-controlled asthma [25] (see Table 1 for more details).

### 3.4. Impact of Uncontrolled Asthma on Daily Life and Health-Related Outcomes

For the included studies, we found that asthma has a significant impact on the daily lives of Saudi adults, as evidenced by various studies uncontrolled asthma can be linked to education, employment status, income level, gender, age, regional, environmental factors, tobacco use, hospitalization, quality of life, adherence to medications, and asthma symptoms or exacerbations [12,19,20] (see Table 2 for more details).

- Education

Education relating to controlling asthma symptoms was discussed in seven studies [8,12,14,15,16,19,21]. Ahmed A.E. (2014) highlighted that asthma control scores varied significantly with the severity of asthma, emphasizing the importance of education about asthma medication and disease. Specifically, participants who received education about asthma medication had slightly better control scores (17.7 ± 3.6) compared to those who did not (17.4 ± 3.9) [12]. Similarly, those who received education about asthma disease or have higher education degree had control scores 3.1 times (OR 3.1) higher compared to those who did not [12,21]. Al-Jahdali et al. also emphasized that higher education levels and regular follow-up with clinics were crucial for better asthma control [8,14,15,16]. For instance, in a 2012 study, regular ICS use was associated with better control (80.6% vs. 72.4%), and follow-up with clinics showed better control (77.8% vs. 75.1%). Poor asthma control was often linked to improper use of inhalers and a lack of education, with 45% of participants in a 2013 study using inhaler devices improperly [16]. Additionally, Al-Zahrani J.M. et al. (2015) reported that uncontrolled asthma was more prevalent among individuals with lower education levels and those who were unemployed. For instance, 39.8% of patients had uncontrolled asthma, with improper device use being more frequent among those with uncontrolled asthma (64.2% vs. 35.8%) [19].

- Employment status

With regards to employment status, two studies found that unemployment is associated with higher rates of uncontrolled asthma [19,21]. Al-Zahrani J.M. et al. (2015) reported 39.8% of unemployed patients had uncontrolled asthma, while Alzayer et al. (2022) highlighted that unemployed, disabled, or ill patients had higher odds (OR 3.1) of uncontrolled asthma [19,21].

- Income level

Torchyan et al. (2017) found that a higher monthly household income was associated with better asthma-related quality of life (AQL), with a monthly income of SAR 25,000 or more linked to improved AQL among men [26]. Al-Jahdali et al. (2019) reported that patients without medical insurance coverage were more likely to have controlled asthma, highlighting the crucial role of access to healthcare resources, which can be influenced by income level, in asthma control [8].

- Gender differences

Based on the included studies, there are notable gender differences in asthma control in six studies [8,13,14,17,20,26]. Al-Ghamdi et al. (2019) reported that in the Aseer Region, the prevalence of wheezing in the past 12 months was higher in females (21.5%) compared to males (18.3%) [13]. Al-Jahdali et al. (2008) found that uncontrolled asthma was prevalent among both genders, but the data did not specify a significant difference between males and females [14]. However, Al-Jahdali et al. (2019) highlighted that a higher level of asthma control was reported among male patients compared to females, with the mean Asthma Control Test (ACT) score being 17.1 (±4.6) for the overall population [8]. Torchyan et al. (2017) found no statistically significant difference in asthma-related quality of life (AQL) between males and females, with mean AQL scores of 4.3 (SD = 1.5) for males and 4.0 (SD = 1.3) for females (*p* = 0.113) [26]. Alomary et al. (2022) indicated that 56.9% of the participants were males, but did not provide specific data on gender differences in asthma control [17]. Similarly, Alzahrani et al. (2024) involved 151 patients, with 23.8% males and 76.2% females, but did not specify gender differences in asthma control, noting instead that most participants did not smoke [20].

- Age and regional variation

Five studies highlight age-related variations in asthma control across different studies in Saudi Arabia. Al-Jahdali et al. (2008) [14] conducted a study in Riyadh and found that younger age groups had better asthma control compared to older age groups. Specifically, the prevalence of uncontrolled asthma was lower in participants under 20 years old (50%) compared to those aged 20–39 (66%), 40–60 (66%), and over 60 (65%). However, other studies reported that age did not significantly affect asthma control, with similar rates of uncontrolled asthma across different age groups [8,15,23,25].

- Environmental factors

Two of the included studies revealed that environmental and lifestyle factors also play a crucial role in asthma management. Al-Ghamdi et al. (2019) indicated that exposure to both outdoor and indoor aeroallergens, such as ragweed and dust mites, was higher among asthmatics [13]. For example, 24.5% of asthmatics had positive specific IgE antibodies to ragweed compared to 20.5% of non-asthmatics [13]. Additionally, living near heavy traffic, having pets, and using analgesics were identified as significant risk factors for asthma. The study found that living near heavy truck traffic increased the risk of asthma (aOR = 1.67), and having cats in the house was also a significant factor (aOR = 2.27) [13]. Alomary et al. (2022) [17] further supported these findings, showing that tobacco use, exposure to moisture, and heating the house were associated with increased wheezing and asthma symptoms. Specifically, daily tobacco use was associated with wheezing (aOR 2.7) [17].

- Tobacco Use

The relationship between tobacco use and asthma control has been discussed in five included studies, revealing significant associations between smoking and poor asthma outcomes. Al-Jahdali et al. (2012) found that active smoking was strongly linked to uncontrolled asthma, with 52.2% of active smokers experiencing uncontrolled asthma compared to 47.8% of non-smokers [15]. Similarly, Alomary et al. (2022) indicated that daily tobacco use was significantly associated with wheezing, with an adjusted odds ratio (aOR) of 2.7 (95% CI: 2.0–3.5), highlighting the impact of smoking on respiratory health [17]. BinSaeed (2015) further demonstrated that daily tobacco smokers had a higher prevalence of uncontrolled asthma (85%) compared to those who smoked less frequently or not at all (67.2%) [6]. Additionally, Torchyan et al. (2017) found that daily tobacco smoking among males was associated with a decrease in asthma-related quality of life (AQL) by 0.72 points (95% CI: −1.30 to −0.14) [26]. Women who had a household member smoking inside the house also had a significantly lower AQL (B = −0.59, 95% CI: −1.0 to −0.19) [26]. However, Al-Jahdali et al. (2019) reported no significant difference in asthma control levels between non-smokers, active smokers, and past smokers (*p* = 0.824), suggesting that other factors may also play a role in asthma management [8].

- Emergency Department Visits and Hospitalizations

Four included studies underscored the relationship between uncontrolled asthma and healthcare utilization, particularly in terms of emergency department (ED) visits and hospitalizations. Ahmed A.E. (2014) found a strong association between frequent ED visits and poor asthma control, with participants experiencing poor asthma control requiring more frequent doctor and hospital visits [12]. Specifically, the study reported that patients with fewer than three ED visits had an average asthma control score of 18.0 (±3.6), while those with three or more ED visits had a lower average score of 16.6 (±3.6) [12]. Similarly, Al-Jahdali et al. (2012) reported that patients with uncontrolled asthma had more frequent ED visits compared to those with controlled asthma [15]. Additionally, Al-Jahdali et al. (2013) linked uncontrolled asthma to higher rates of hospitalizations due to asthma exacerbations [16]. Tarrafa H et al. (2018) further supported these findings, noting that frequent nighttime symptoms and exacerbations affecting daily activities and sleep were prevalent among patients with uncontrolled asthma, leading to increased hospital admissions [24].

- Quality of Life

The quality of life (QoL) and psychological well-being of asthma patients were discussed in four included studies. Torchyan et al. (2017) demonstrated that uncontrolled asthma is associated with a lower asthma-related quality of life (AQL), with daily tobacco smoking among males decreasing AQL by 0.72 points (95% CI: −1.30 to −0.14) [26]. This study also found that women who had a household member smoking inside the house experienced a significantly lower AQL (B = −0.59, 95% CI: −1.0 to −0.19), indicating the broader impact of second-hand smoke on asthma patients [26]. Al-Jahdali et al. (2019) corroborated these findings, showing that patients with controlled asthma had better QoL according to the SF-8 questionnaire [8]. Furthermore, Alzahrani et al. (2024) linked uncontrolled asthma to higher levels of frustration and fear of not having asthma medication, with 12% of patients feeling afraid all the time and 13% feeling frustrated some of the time [20]. Alzayer et al. (2022) found that poorly controlled asthma was associated with lower scores on the Asthma Control Test (ACT), indicating a negative impact on mental health [21].

- Adherence to Medication

Adherence to medication including follow-up with healthcare providers, particularly inhaled corticosteroids (ICSs), was reported in three included studies. Al-Jahdali et al. (2012) demonstrated that regular use of ICSs was associated with better asthma control, with 80.6% of patients who regularly used ICSs having partially or fully controlled asthma compared to 72.4% of those who did not use ICSs regularly [15]. Al-Jahdali et al. (2013) also highlighted the impact of improper use of inhaler devices on asthma control, linking it to higher rates of uncontrolled asthma, with 45% of patients using inhaler devices improperly [16]. Regular follow-up with healthcare providers is another critical factor, as Ahmed AE (2014) found that patients who consistently followed up with their doctors had better asthma control, with an average asthma control score of 17.4 (±3.4) for those who followed up regularly compared to 17.8 (±4.2) for those who did not [1]. Al-Jahdali et al. (2012) supported this finding, showing that patients who regularly attended clinic visits had better asthma control compared to those who did not [15].

- Asthma symptoms

Only one study, by Ghaleb Dailah (2021), provided insights into asthma control levels and symptom frequency among the participants. The study found that the majority of the control group had somewhat controlled asthma (38.1%), while completely controlled asthma was observed in 23.8% of the control group. Additionally, poorly controlled asthma was present in 11.1% of the participants. The study also highlighted the frequency of asthma symptoms, with 27% of participants experiencing symptoms such as wheezing, coughing, shortness of breath, and chest tightness or pain once or twice a week. Furthermore, 31.7% of the participants reported using rescue inhalers or nebulizers (such as albuterol) two or three times per week. These findings underscore the varying levels of asthma control among the participants and the regular occurrence of asthma symptoms and medication use [22]. Symptoms reviewed in the included studies cough [22], wheezing [13,17,22], shortness of breath [20,22,25], sleep disturbance or sleep quality [15,20,24], and limited daily activities [24,25].

- Meta-analysis

The meta-analysis revealed an overall odds ratio of 1.23 (95% CI: 0.98–1.54), suggesting a slight but statistically non-significant association between asthma control status and the examined factors. The unadjusted analysis yielded a broader range with an odds ratio of 0.11 (95% CI: 0.07–6.58), indicating high variability across studies (refer to Supplementary Materials, pooled analysis for the meta, for additional details).

## 4. Discussion

The aim of this systematic review is to comprehensively assess the literature on the significant burden of uncontrolled asthma among the Saudi Arabian population, with particular attention to contributing factors such as education level, environmental exposure, and treatment adherence. This review seeks to deepen the understanding of studies that examine factors and conditions influencing asthma control and management. Specifically, it focuses on the prevalence and impact of uncontrolled asthma, as well as key determinants that affect disease control. Investigating the prevalence of uncontrolled asthma in Saudi Arabia and identifying its contributing factors is essential for developing effective management strategies and improving patient outcomes. In particular, understanding the role of socioeconomic and environmental influences is critical for shaping public health policies aimed at reducing disparities in asthma prevalence and outcomes across regions of the Kingdom. Furthermore, examining these factors promotes a comprehensive approach to asthma management that extends beyond pharmacological treatment. It encourages healthcare systems to consider the broader context of patients’ lives, including education level, living conditions, and social support networks—elements that ultimately contribute to better quality of life and improved asthma outcomes. Overall, our findings underscore the urgent need for targeted interventions, including patient education, improved access to inhaled corticosteroids (ICSs), and strategies to reduce environmental triggers, in order to enhance asthma control and optimize patient care in Saudi Arabia.

Understanding the prevalence of asthma control is essential as it reflects the effectiveness of current management strategies and highlights variations in outcomes across different patient demographics [28,29]. This, in turn, can inform public health initiatives and guide resource allocation to optimize disease control. Analysis of the six studies reporting asthma symptom prevalence in Saudi Arabia reveals that approximately 68% of patients with asthma experience uncontrolled symptoms across the Kingdom. Moreover, our review finds that poor asthma control significantly impairs patients’ daily functioning.

The included studies consistently demonstrate a negative association between asthma control and factors such as education level, employment status, gender, age, medication adherence, tobacco use, quality of life, and frequency of emergency visits. In contrast, a positive correlation is observed between medication adherence and improved asthma symptom control. These findings align with previous research indicating that poor asthma control adversely impacts patient well-being, leading to more frequent exacerbations, increased healthcare utilization, and reduced quality of life [30]. Additionally, a study by Backman et al. (2019) showed that inadequate asthma control can worsen health disparities and impose substantial economic burdens on affected populations [31]. Overall, these findings underscore the urgent need to improve asthma management in Saudi Arabia to mitigate these outcomes and enhance patient care.

Our study suggests that awareness of asthma, level of education, employment status, and income level are all associated with the prevalence of asthma control in Saudi Arabia. A study by Nguyen et al. (2018) found that patients with higher education levels demonstrate better understanding of asthma management and experience improved disease outcomes, whereas patients with lower educational attainment face difficulties comprehending information provided by healthcare professionals [32]. Furthermore, enhancing patient awareness of asthma and its medications has been shown to improve medication adherence by 80%, leading to reduced emergency department visits and hospital admissions [16,33]. Additional studies have also shown that patients with stable employment status tend to have better asthma control [34]. Similarly, our findings indicate that patients with higher education levels, stable employment, and greater knowledge about asthma achieve better outcomes than their counterparts. These findings underscore the importance of asthma education, which can be effectively delivered through asthma clinics. Such clinics can play a critical role in improving patients’ knowledge and equipping them with essential self-management skills, ultimately leading to improved asthma control. Addressing disparities in health education may also promote better asthma outcomes and contribute to broader public health improvements, including enhanced job stability, increased income, and improved overall quality of life [35].

Our systematic review demonstrates that patients with uncontrolled asthma symptoms in Saudi Arabia experience lower asthma-related quality of life (QoL). The impact of daily tobacco smoking and passive smoke exposure on asthma control was inconclusive: two studies reported that both active and passive smoking negatively affected asthma-related quality of life (AQL), while another study found no significant association. These findings are consistent with previous research showing that active smokers with asthma experience higher rates of exacerbation and significantly poorer asthma-related quality of life compared to non-smokers [36]. Similarly, Mroczek et al. (2015) reported that low asthma control is associated with increased treatment costs and reduced health-related quality of life (HRQoL) [37]. Overall, these results emphasize the importance of effective asthma management strategies and the implementation of smoking cessation programs to improve patient outcomes and reduce the burden of disease.

The management and prevalence of asthma symptoms and exacerbations are influenced by multiple factors, including gender, age, environmental exposures, and regional variations [38,39,40,41,42]. Gender in particular plays a significant role in asthma prevalence. A study by Almqvist et al. (2008) reported that asthma is more prevalent in boys than girls during childhood, while the trend reverses during adolescence, with women exhibiting higher rates than men [38]. Similarly, our review identified notable gender differences, with asthma being more frequently reported among women. Moreover, the analysis indicated that men with asthma generally had better symptom control compared to women.

Environmental and regional factors also significantly affect asthma symptoms and the frequency of exacerbations [43,44]. Studies have shown that patients living near factories, in areas with poor air quality, or in agricultural regions, as well as those with high exposure to dust mites, mold, or pollen are more likely to experience asthma exacerbations and have lower levels of asthma control [45,46]. These findings are consistent with our review, which notes associations between increased exposure to environmental triggers—such as ragweed, moisture, dust mites, and heavy traffic—and a higher prevalence of symptoms like wheezing. These results underscore the importance of evaluating a wide range of contributing factors when designing asthma management strategies. Enhancing patient awareness of environmental triggers and expanding clinical support services—particularly in regions with high exposure to such triggers—can play a vital role in improving asthma care in Saudi Arabia. Furthermore, implementing routine asthma screenings in educational institutions may improve early identification and long-term disease control, ultimately enhancing patient outcomes.

Adherence to medication, asthma symptoms, and emergency department (ED) visits have been widely associated with asthma severity and patients’ quality of life [47,48,49,50,51]. Our review finds that asthma control is positively influenced by adherence to medication and negatively affected by frequent symptoms and ED visits. Specifically, patients who adhere to inhaled corticosteroid (ICS) therapy and attend regular follow-ups demonstrate better asthma outcomes. Additionally, our findings show that patients who experience asthma symptoms more than 2–3 times per week and rely on rescue inhalers more than twice per week often report limited daily activity and sleep disturbances. Furthermore, poor asthma control is associated with increased rates of ED visits and hospital admissions. Our review also indicates that patients with uncontrolled asthma have lower scores on both the Asthma Quality of Life Questionnaire (AQLQ) and the Asthma Control Test (ACT), alongside reported emotional burdens such as frustration and fear related to medication availability. These findings highlight the critical importance of effective asthma management, which can be supported through routine assessments such as pulmonary function tests (PFTs) and blood tests to evaluate asthma severity. Increasing patient awareness of medication usage and ensuring access to asthma therapies are essential steps toward reducing exacerbation frequency, improving quality of life, and lowering overall healthcare costs.

## 5. Limitations

This systematic review provides valuable insights into asthma control among residents of Saudi Arabia; however, several limitations should be acknowledged. First, there was considerable heterogeneity in the study samples, methodologies, and reported outcomes across the included studies, which may affect the comparability of findings. Second, this review did not include adolescent populations—a demographic that may exhibit different patterns or disparities in asthma control outcomes. Despite these limitations, this systematic review makes a meaningful contribution to the growing body of knowledge on the prevalence and determinants of asthma control in Saudi Arabia.

## 6. Conclusions

Asthma control among adults in Saudi Arabia remains a significant public health concern. Despite the availability of effective treatment options and national clinical guidelines, many patients continue to experience poor disease control due to factors such as limited awareness, medication non-adherence, socioeconomic status, employment conditions, age, and environmental triggers. Improving asthma outcomes requires a multifaceted approach that includes patient education, regular follow-up visits (including pulmonary function tests [PFTs] and asthma severity assessments), individualized treatment plans, and broader public health initiatives aimed at reducing exposure to allergens and pollutants. Strengthening primary care services and implementing nationwide asthma management programs can play a critical role in enhancing disease control and improving quality of life for adults living with asthma in Saudi Arabia. Additionally, further research is recommended to better understand the factors influencing asthma control and to inform evidence-based interventions tailored to the Saudi context [52].

## Figures and Tables

**Table 1:** Summary of Included Studies on Asthma Control Among Adults in Saudi Arabia.

Author (Year)	Design	Location	Sample Size	Key Findings	Asthma Diagnosis
(Ahmed A.E., 2014) [12]	Cross-sectional study	Riyadh, Saudi Arabia	450 participants	Asthma control levels: ○ Controlled 17.5%. ○ Uncontrolled 19%. Poor asthma control was associated with: ○ Older age. ○ Frequent ED visits (≥3/year). Better control was linked to: ○ Patient education on asthma medication and disease. Asthma control scores declined with increasing disease severity: ○ Severe asthma had the lowest scores (mean = 8.5). ○ Poor control correlated with reduced quality of life and higher healthcare use.	Clinically diagnosed
(Al-Ghamdi et al., 2019) [13]	Cross-sectional study	Aseer Region, Southwestern Saudi Arabia	960 participants	Bronchial asthma (BA) prevalence: 19.2% among adults. No significant gender difference in prevalence (males: 18.3%, females: 21.5%). Significant risk factors for BA: Living in low-altitude areas. Rural residence. Analgesic use. Living near heavy truck traffic. Having cats at home. Age 55–64 years.	Clinically diagnosed
(AL-Jahdali et al., 2008) [14]	Cross-sectional study	Riyadh, Saudi Arabia	1060 participants	Estimated Asthma Prevalence = 4%. Asthma Control level: Uncontrol: 64%, Controlled: 31%, Completely controlled: 5%. No significant difference in asthma control between age groups below and above 40 years. Better control observed in patients younger than 20 years. Gender difference: 44% of males vs. 30% of females had controlled asthma (*p* = 0.0001). Education impact: 71% of those without formal education had uncontrolled asthma (*p* = 0.001).	Clinically diagnosed
(AL-Jahdali et al., 2012) [15]	Cross-sectional study	Riyadh, Saudi Arabia	450 participants	Estimated Asthma Prevalence = 20–25%. Asthma control levels: ○ Uncontrolled= 23.4% (ACT score ≤ 15). ○ Partially controlled= 74.4% (16 ≤ ACT score ≤ 23). ○ Complete controlled asthma= 1.8% (ACT score ≥ 24). Frequent ED visits (≥3/year) were significantly associated with: ○ Uncontrolled asthma (*p* = 0.0003). ○ Lack of asthma education (*p* = 0.0145). Uncontrolled asthma was more common among: ○ Patients with irregular ICS use (27.6%) vs. regular users (19.4%). ○ Those without asthma education (28.1%) vs. those with education (18.1%).	Physician diagnosis
(Al-Jahdali et al., 2013) [16]	Cross-sectional study	Riyadh, Saudi Arabia	450 participants	Estimated Asthma Prevalence = 20–25%. Asthma control levels: ○ Uncontrolled asthma (ACT ≤ 15): 23.3%. ○ Partially controlled asthma (ACT 16–23): 74.4%. ○ Fully controlled asthma (ACT ≥ 24): 1.8%. ○ Missing ACT data: 0.5% Improper inhaler use was observed in 45% of patients. Uncontrolled asthma (ACT ≤ 15) was strongly linked to inhaler misuse (59.1% of these patients used inhaler incorrectly). Factors associated with higher inhaler misuse: ○ Irregular clinic follow-up (60.9%). ○ No education about asthma disease (57.4%). ○ No education on medication/inhaler use (54.6%). ○ Frequent ED visits (≥3/year) (50.9%). ○ Recent asthma diagnosis (<1 year) (77.8%).	Physician diagnosis
(Al-Jahdali et al., 2019) [8]	Cross-sectional study	Saudi Arabia	1009 participants	Estimated Asthma Prevalence = 4–25%. Asthma control levels: 30.1% controlled, 38.0% uncontrolled, partly controlled: 31.9%. ○ Better control associated with: ○ Higher education. ○ Use of ICS + LABA. ○ Females were less likely to have controlled asthma. ○ Controlled asthma was linked to better quality of life. No significant link between control and age, BMI, or smoking.	Physician diagnosis
(Alomary et al., 2022) [17]	Cross-sectional study	Saudi Arabia	7955 participates	Estimated Asthma Prevalence = 17.6%. Wheeze prevalence: 14.2%, highest in ages 20–29. Severe symptoms present in 38.1% of those with wheeze. Asthma diagnosis reported by 14%; 83.3% confirmed by a doctor. Low asthma action plan coverage (38.4%) despite high attack rate (50.5%). Women had more control plans and asthma attacks than men. Common symptoms: sleep disturbance (62.8%), breathlessness (59.4%), and speech limitation (21.6%).	Diagnosed by doctors (83.3%)
(Alqahtani, J.M., 2020) [18]	Cross-sectional study	Najran, southwestern Saudi Arabia	222 participants	Estimated Asthma Prevalence = 4.1–23%. Asthma prevalence among Saudi young adults: 27%, significantly higher in males (*p* = 0.01). Other allergic conditions: ○ Atopic dermatitis: 13.1%. ○ Allergic rhinitis: 5%. ○ Atopy prevalence: 40.5%; over half had symptoms. Gender differences: Males had more respiratory symptoms and were more sensitized to cat hair and ragweed. Females were more sensitized to dog hair and Bermuda grass. Most common allergens: Bermuda grass (20.8%), cat fur (18.9%), dust mites (12.7%).	Physician diagnosis
(Al-Zahrani J.M. et al., 2015) [19]	Cross-sectional study	Riyadh, Saudi Arabia	400 participants	Uncontrolled asthma affected ~40% of patients. Over half misused inhaler devices. Risk factors included smoking, inhaler misuse, and workplace/environmental triggers. Women, unemployed, and single/divorced patients were more likely to have poor control. Despite high physician education rates, misconceptions and poor medication practices were common.	Physician-diagnosed asthma
(Alzahrani et al., 2024) [20]	Cross-sectional study	Al-Baha, Saudi Arabia	151 participants	Asthma control levels: controlled 25.8%, uncontrolled 74.2%. Low quality of life reported by 74.2% of asthma patients (mean age: 52 years). Common triggers: cigarette smoke (69%) and dust (61%). Emotional concerns included fear of medication unavailability (54%) and concern about asthma (43%). Notable activity limitations in both social and work life. Factors like age, gender, smoking, and chronic diseases showed weak correlation with quality-of-life scores.	Symptoms report
(Alzayer et al., 2022) [21]	A qualitative research method	Riyadh, Saudi Arabia	20 participant	Most participants were young, educated women (avg. age 32). Over half had well-controlled asthma (ACT ~19). Patients expressed: Fear, stigma, and low medication knowledge. Preference for herbal remedies. Trust in doctors was high; pharmacists seen as less involved. Family and social media played key roles in asthma support and decisions.	Interviews
(BinSaeed, 2015) [6]	Cross-sectional survey	Riyadh, Saudi Arabia	260 participants	Uncontrolled asthma was common (68.1%) among relatively young adults (avg. age: 32). Associated factors: ○ Tobacco use, low income, and comorbidities (e.g., sinusitis, heartburn). ○ Older age (≥35) and female gender doubled the odds of poor control. ○ Lower education and unemployment also linked to worse control. ○ High rates of allergic rhinitis (66%) and chronic sinusitis (39%) were observed.	Patients had been diagnosed by a physician at least 3 months before joining the stud
(GhalebDailah, 2021) [22]	Cross-sectional study	Saudi Arabia, Jeddah and Jazan	263 participants	Asthma control levels: 38.1% controlled, 23.8% completely controlled, 11.1% poorly controlled. Intervention group showed clear improvements in: ○ Asthma self-management. ○ Knowledge. ○ Patient activation. Control group showed minimal or no improvement across all measures.	Clinically diagnosed
(Habib et al., 2014) [23]	Cross-sectional study	Riyadh, Saudi Arabia	53 participants	54.7% had poorly controlled asthma (ACT < 20); 45.3% were well-controlled. Most poorly controlled patients (79.3%) had elevated FeNO. FeNO levels were significantly higher in poorly controlled asthma (65.5 ppb) vs. well-controlled (27.4 ppb), *p* < 0.001. Strong negative correlation between FeNO and ACT score (r = −0.581).	Clinically diagnosed
(Tarrafa H et al., 2018) [24]	Cross-sectional study	11 Middle Eastern and North African Countries including (Saudi Arabia)	7236 participants	Asthma control levels: ○ Controlled: 29.4%. ○ Uncontrolled: 45.0%. Poor medication adherence: Only 22.9% reported good adherence (Morisky score). Exacerbations impacted 18.5% of patients’ daily life and sleep. Most patients (97.1%) were receiving treatment: ○ 62.7% used ICS + LABA + other therapies. ○ 13.6% used ICS + LABA only. Quality of life scores were modest (Physical: 43.0, Mental: 44.9).	Clinically diagnosed and patient assessment
(Tayeb et al., 2017) [25]	Cross-sectional study	Jeddah, Saudi Arabia	173 participants	Asthma control levels: ○ Uncontrolled: 63%. ○ Partially controlled: 34%. ○ Controlled: 3%. Use of asthma-triggering drugs (ATDs): ○ 51% were using ATDs; most had uncontrolled or partially controlled asthma. ○ Even among those not using ATDs, uncontrolled asthma remained high (32%). Polypharmacy: ○ Uncontrolled patients used an average of 4 ATDs. ○ Common ATDs: Aspirin, ACE inhibitors, NSAIDs, β-blockers.	Clinically diagnosed
(Torchyan et al., 2017) [26]	Cross-sectional study	Riyadh, Saudi Arabia	257 participants	Asthma control levels: ○ Controlled 32.2%. Asthma Quality of Life (AQL) scores were slightly higher in men (4.3) than women (4.0). Better asthma control was consistently linked to improved AQL in both genders. Negative AQL factors: ○ Daily smoking (men: −0.72 points). ○ Household smoking exposure (women: −0.59 points). Positive AQL predictors: ○ Higher income (men: +0.51). ○ Employment (women: +0.48). ○ Perceived asthma severity (women: +0.82).	Clinically diagnosed based on medical history

Abbreviations: CS, cross-sectional; RCT, randomized controlled trial; NA, not available; ACT, Asthma Control Test; GINA, Global Initiative for Asthma; FEV_1_, forced expiratory volume in one second; PFT, pulmonary function test; QoL, quality of life; AQLQ, Asthma Quality of Life Questionnaire; PAQLQ, Pediatric Asthma Quality of Life Questionnaire (if applicable); BDI, Beck Depression Inventory; HADS, Hospital Anxiety and Depression Scale; ER, emergency room; ED, emergency department; ICS, inhaled corticosteroids; LABA, long-acting beta agonist; OR, odds ratio; CI, confidence interval; KSA, Kingdom of Saudi Arabia.

**Table 2:** Prevalence of Asthma Control and Associated Factors Reported in Included Studies.

Author (Year)	Age (Years)	(Sex M/F)	Symptoms Control	ICS Used?	Asthma Symptoms	Medication Regimen	Impact of Asthma on Quality of Life	Impact of Tobacco Use on Asthma Control	Impact of Educational Level on Asthma Control	Other Factors That May Impact Asthma Control
(Ahmed AE, 2014) [12]	42.3 ± 16.7 years	176 (39.1%) male and 274 (60.9%) female	Significant difference in the asthma control scores for severe persistent asthma: - (M = 8.5, SD = 2.0) - mild persistent (M = 18.0, SD =2.3) - intermittent asthma (M = 19.3, SD = 3.1)	50.4% of the participants used ICS	14 (3.1%) participants were considered to have severe persistent asthma, 75 (16.7%) participants were moderate persistent, 181 (40.2%) participants were mild persistent, and 180 (40%) participants were mild intermittent	▪ Half of the Participants, 203 (45.1%), use asthma device improperly ▪ 266 (59.1%) claim that they received education about asthma medications ▪ 50.4% of the participants used ICS	There is an association between frequent ED visits and poor asthma control from pervious studies. Participants with poor asthma control have poor health-related quality of life, more doctor and hospital visits	NA	Study did not find that demographic factors such as gender, marital status, and level of education or job status were responsible for poor asthma control as defined by ACT	One possible explanation is probably that our patients have free access to hospitals and free dispensing of asthma therapy, which probably limits the influence of job status and income as a factor for poor asthma control
(AlGhamdi et al., 2019) [13]	Adult (≥20 years of age)	NA	Persons with an increase in total IgE (>100 IU/mL) had significantly higher probability (OR = 1.84, 95% CI: 1.10–3.06) to develop adult asthma. Similarly, those with an increase in total peripheral Eosinophil count (>150 cells/mm^3^ ) had more than two times the risk to have adult asthma (OR = 2.85, 95% CI: 1.14–7.15)	NA	NA	NA	NA	NA	NA	Rye wheat is an important outdoor sensitization factor for bronchial asthma in adults
(AL-Jahdali et al., 2008) [14]	Median age was 38.56 years (range 15–75)	Total number of patients studied was 1060. Males comprised 442 (42%) and females comprised 618 (58%)	ACT score revealed uncontrolled asthma in 667 (64%), well-controlled asthma in 383 (31%), and completely controlled in 55(5%)	NA	NA	NA	Of the major reasons for poor asthma control is poor compliance	NA	Significant correlation between level of education and asthma control, 71% of patients who did not have formal education had uncontrolled asthma (*p* = 0.001)	Younger age group (less than 20 years old) had better asthma control compared to the older age group (*p* = 0.0001)
(AL-Jahdali et al., 2012) [15]	42.3 ±16.7 years	M = 176 (39.1%), F = 274 (60.9%)	NA	Partially/Fully controlled (n = 343) Level (ACT) - Regular ICS use: Yes (80.6) No (72.4) Not controlled (n = 105) Level (*p*-Value) - Regular ICS use: Yes (19.4) No (27.6)	NA	NA	Frequent emergency department visits	NA	Partially/Fully controlled (n = 343) Level (ACT) - Education level: high school or less (77.2), university (72.1) Not controlled (n = 105) Level (*p*-Value) - Education level: high school or less (22.8), university (27.9)	-Lack of education about asthma -Treatment needs (patients visited ED primarily to receive a bronchodilator by nebulizer and oxygen) - Inadequate use of ICS
(Al-Jahdali et al., 2013) [16]	42.3 ±16.7 years	M = 176 (39.1%) F = 274 (60.9)	NA	The study mentions that roper use of ICS therapy is essential for effective asthma control and reducing the likelihood of uncontrolled asthma and frequent ED visits	NA	- MDI: 361 (80.2) - Turbuhaler: 43 (9.6) - Diskus: 38 (8.4) - MDI with spacer: 3 (0.7)	NA	NA	NA	NA
(Al-Jahdali et al., 2019) [8]	- 48.7 years (±15.9) - 18 to 35 = 222 (22) - 35 to 55 = 425 (42.1) - 55 to 70 = 260 (25.8) - 70 and above = 102 (10.1)	M = 350 (34.7) F = 659 (65.3)	NA	- Inhaled corticosteroids: 197 (19.6) - Patients using fixed combination (inhaled corticosteroids + long-acting beta-agonist) and those using antileukotrienes were more likely to have controlled asthma compared to patients not taking such medications (OR: 1.77 [95% CI: 1.29–2.44] and OR: 2.39 [95% CI: 1.82–3.14], respectively)	NA	Inhaled corticosteroids: 197 (19.6) Long-acting bronchodilator: 90 (9.0) Oral corticosteroids: 76 (7.6) Fixed combination (inhaled corticosteroids + long-acting beta-agonist): 833 (82.9) Antileukotrienes: 367 (36.5) Theophylline: 55 (5.5) Anticholinergic bronchodilator: 96 (9.6) Short-acting beta-agonist: 546 (54.3) Nasal corticosteroids: 41 (4.1) Antihistamine: 12 (1.2)	Patients with controlled asthma had better QoL according to SF-8 questionnaire (*p* < 0.001), but they did not show better medication adherence (according to MMAS-4© score)	Nonsmokers did not show any significant difference in asthma control levels when compared to active smokers and past smokers (*p* = 0.824)	Patients with higher educational level were almost four times more likely to have controlled asthma (OR: 3.72 [95% CI: 1.74–7.92])	Patients without medical insurance coverage were more likely to have controlled asthma (OR: 1.44 [95% CI: 1.09–1.90])
(Alomary et al., 2022) [17]	The mean participant age was 38.6 years	56.9% were men	NA	NA	Wheeze: 882 (14.2%)	NA	NA	Using tobacco daily was associated with wheezing (aOR 2.7; 95% CI: 2.0–3.5)	NA	- Significant factors associated with wheeze were: - jobs (aOR 11.8; 95% CI: 7.3–18.9) - exposure to moisture or damp spots (aOR 2.2; 95% CI: 1.5– 3.4) - heating the house when it is cold (aOR 1.7; 95% CI: 1.3–2.1)
(Alqahtani, J.M., 2020) [18]	19 to 23 (21.5 ± 1.5) years	M = 116 students, F = 106 students were included	NA	NA	“Asthma” Participants without atopy (N= 122) - Wheeze “ever”: 25 (20.4) - Current wheeze: 13 (10.7) - Physician-diagnosed BA: 26 (21.3) - Exercise-induced asthma: 28 (25) - Nocturnal cough: 42 (38.2) Participants with atopy (N= 90) - Wheeze “ever”: 40 (44.4) - Current wheeze: 32 (35.6) - Physician-diagnosed BA: 34 (37.8) - Exercise-induced asthma: 22 (24.4) - Nocturnal cough: 28 (31.1)	NA	NA	NA	NA	NA
(Al-Zahrani J.M. et al., 2015) [19]	Adults (≥18 years of age)	The sample included 120 males (30%) and 280 females (70%)	Uncontrolled asthma was defined as an ACT score ≤ 16. Findings show that 39.8% of patients had uncontrolled asthma	NK	NK	A majority of patients used bronchodilators as their main inhaler, and 72.2% used it only for asthma therapy. Findings revealed that 55.2% were using the meter-dosed inhaler as their main device	NK	Active smoking (*p*-value = 0.007), passive smoking (*p*-value = 0.019	Approximately half of the patients had received a high school education or less, 38.9% had no education, and only 12% had university education. Unemployment was significantly associated with uncontrolled asthma (*p*-value = 0.019)	Improper device use by the patient was more frequently associated with uncontrolled asthma (46.9% partially/fully controlled vs. 64.2% uncontrolled asthma, *p*-value = 0.001)
(Alzahrani et al., 2024) [20]	Adults (≥18 years of age)	The sample included 36 males (23.8%) and 115 females (76.2%)	NK	NK	- Environment-related symptoms - Emotion-related symptoms	NK	The present findings indicate the considerable influence of asthma on quality of life	Most of the participants did not smoke (91.4%	NK	Among the participants, 78 individuals (51.7%) had chronic diseases in addition to asthma
(Alzayer et al., 2022) [21]	Adults (≥18 years of age)	The sample included 4 males (17%) and 19 females (82%)	Participants’ asthma control scores indicated that 52% (n = 12) of participants or those with asthma they cared for had only partially controlled asthma (ACTTM score < 19), while 13% (n = 4) had poorly controlled asthma (ACTTM score < 15)	NK	NK	NK	NK	NK	Patients with less than a graduate degree had a 3.1-fold higher likelihood of experiencing uncontrolled asthma (OR = 3.1; 95% CI: 1.0–9.5). Similarly, those who were unemployed, disabled, or too ill to work exhibited significantly greater odds of having uncontrolled asthma (OR = 3.1; 95% CI: 1.4–6.9). These findings are consistent with existing literature indicating that education level and occupational status are important determinants of asthma control	Findings clearly highlighted lack of knowledge about the role of different types of asthma medications. Most participants were rather unclear, for example, about the differences between reliever and preventer medications
(BinSaeed, 2015) [6]	Adults (≥18 years of age)	The sample included 130 males (50.0%) and 126 females (48.8%)	The proportion of patients with uncontrolled asthma in our study population was 68.1%	NK	Experiencing heartburn symptoms within the past four weeks was linked to a 2.5-fold increase in the odds of having uncontrolled asthma (OR = 2.5; 95% CI: 1.3–4.9)	NK	NK	It shows that tobacco smoker who smoke daily have uncontrolled asthma 17/20 (85%). On the other hand, tobacco smoker who smoke less than daily or not at all have uncontrolled asthma 156/232 (67.2%)	Individuals with less than a graduate degree had 3.1 times higher odds of experiencing uncontrolled asthma (OR = 3.1; 95% CI: 1.0–9.5). Similarly, those who were unemployed, disabled, or too ill to work also showed a 3.1-fold increased risk of uncontrolled asthma (OR = 3.1; 95% CI: 1.4–6.9). These findings support existing evidence that education level and employment status are key factors associated with asthma control	Bivariate analysis indicated that several factors—including age, gender, marital status, education level, occupation, monthly household income, obesity, chronic sinusitis or allergic rhinitis, and recent heartburn symptoms (within the past four weeks)—were significantly associated with uncontrolled asthma
(GhalebDailah, 2021) [22]	Adults (≥18 years of age)	The sample included males (46%) and females (54%)	Majority of control group have somewhat controlled asthma 24 (38.1%)	NK	Often control groups have asthma symptoms (wheezing, coughing, shortness of breath, and chest tightness or pain) once or twice a week 17 (27%)	Often control groups have rescue inhaler or nebulizer (such as albuterol) 2 or 3 times per week 20 (31.7%)	NK	NK	NK	NK
(Habib et al., 2014) [23]	36.1 ± 14.3 years	Male: 42 Female: 11	ACT score of <20 is correlated with uncontrolled asthma. In this study, 24 cases had an ACT score >20 And 29 cases had an ACT <20	28.3% used steroids 11.3% used a mix of medications	N/A	15.1% did not take any medications 39.6% used bronchodilators 28.3% used steroids 5.7% used leukotriene inhibitors 11.3% used a mix of medications	The impact of asthma on quality of life was not explored in this paper	Smokers were excluded from the study as smoking is known to reduce FENO values	There was no significant correlation of FENO with age, height, weight, asthma duration, and ventilatory function tests. Educational level was not mentioned	The conventional measures of asthma severity do not assess airway inflammation and may not provide optimal assessment for guiding therapy that helps in asthma control
(Tarrafa H. et al., 2018) [24]	18 years or more	Female: 57% Male: 43%	Controlled or partly controlled: 4202 Uncontrolled: 2977	5.8% of the total population used only ICS as main asthma treatment	- Frequent nighttime symptoms 10% of the population - Exacerbation affecting activities and sleep 22.6%	- 38% used fixed ICS+ LABA with other treatment - 27% used fixed ICS + LABA alone - 8.1% used free ICS + LABA - 5.8% used only ICS - 4.5% used SABA alone - 16.6% used other treatments	- Frequent night symptoms were reported in 10.3% of patients - 66.3% of the population had a history of mild exacerbations - 22.3% of patients reported an impact on daily activities and sleep	- 80.1% of the total population were non-smokers - 9.1% were past smokers - 10.8% were active smokers	Patients with a higher level of education were more likely to have controlled asthma (OR, 2.31 (95% CI 1.72, 3.09)	Poor asthma control can be addressed by improving access to appropriate treatments, encouraging better medication adherence and smoking avoidance, along with more proactive follow-up and better education among both healthcare providers and patients
(Tayeb et al., 2017) [25]	Mean age: 44 ± 16 years	Female: 70 Male: 103	63% had uncontrolled asthma 34% were partially controlled 3% had controlled asthma	N/A	The cardinal asthma symptoms are shortness of breath, wheeze, cough, chest tightness	Asthma medications were not mentioned	- Continuous morbidity - Poor productivity - Frequent absence from work - Frequent visits to outpatient clinics and emergency rooms - Financial burden on asthmatics and health systems	N/A	The study reflects the unacceptably low awareness of health professionals about the harmful effects of asthma-triggering drugs on asthma control levels. Regular asthma educational courses for health professionals are important	Asthma-triggering drug use is a substantial cause of poor asthma control. This reflects the low awareness of health professionals about the negative effects of these drugs on asthma control
(Torchyan et al., 2017) [26]	Adults aged 18 years and above	Male: 129 Female: 128	- 67.8% of the total population had uncontrolled asthma - 32.2% had controlled asthma	The use of ICS was not discussed in this paper	Symptoms in the studied population were not discussed clearly in this paper	Asthma medications were not mentioned	- 4.1 mean (1.4 SD) suffered from symptoms - 4.4 (1.5) had activity limitations - 4.3 (1.6) emotional function - 3.9 (1.5) environmental stimuli	Tobacco smoking was associated with 0.72-point decrease (95% CI = A −1.30–−0.14) in the AQL among males. The decreased quality of life might be attributed to increased inflammation in the airways and reduced sensitivity to corticosteroids caused by cigarette smoking	Effect of level of education on asthma control was not explored in this paper	This paper reveals gender-specific differences in the correlates of AQL in Saudi Arabia

Abbreviations: ACT, Asthma Control Test; GINA, Global Initiative for Asthma; QoL, Quality of Life; ICS, Inhaled Corticosteroids; LABA, Long-Acting Beta Agonist; ED, Emergency Department; ER, Emergency Room; OR, Odds Ratio; CI, Confidence Interval; NA, Not Available. The table summarizes prevalence estimates of controlled, partially controlled, and uncontrolled asthma, as well as associated demographic, clinical, behavioral, and environmental factors as reported in each study.

## Data Availability

Data sharing not applicable.

## Supplementary Materials

The following supporting information can be downloaded at: https://www.mdpi.com/article/10.3390/jcm14165753/s1, Figure S1: Asthma control in Saudi Arabia. Table S1: Summary of included studies in the meta-analysis, Calculation of Odd ratio by Sample size. PRISMA 2020 Checklist. Reference [52] is cited in the Supplementary Materials.
