# Supplementary material for: Asthma Control Among Adults in Saudi Arabia: A Systematic Review and Meta-Analysis
Source: J Clin Med. 2025 Aug 14;14(16):5753. doi: 10.3390/jcm14165753 (PMC12386509; doi:10.3390/jcm14165753)
Supplement: Supplementary file 1 [file jcm-14-05753-s001.zip › jcm-3686807-Table S1.pdf]

To Calculated the pooled control and uncontrolled asthma percentage. We consider the study as a unit of analysis and weighted studies by sample size. Studies with larger sample size have more weight in the analysis than studies with sample size. Then, we provide unadjusted analysis without the study weight and the adjusted analysis with the study weight.

This statistical method is valid and reliable and has been used previously in many published papers in respiratory care.

https://www.youtube.com/watch?v=ax8jNMJtdN8

Summary of included studies in the meta-analysis

Author (Year)	Sample Size	UN	CON
AL-Jahdali et al, 2008	1060	64	36
AL-Jahdali et al, 2012	450	23.4	76.6
Al-Jahdali et al, 2013	450	23.4	76.6
Al-Jahdali et al. 2019	1009	38	62
Al-Zahrani JM et al. 2015	400	39.8	60.2
BinSaeed, 2015	260	68.1	31.9
GhalebDailah, 2021	263	27	73
Habib et al., 2014	53	54.5	45.5
Tarrafa H et al. 2018	7306	70.6	29.4
Tayeb et al., 2017	173	63	37
Torchyan et al., 2017	257	67.8	32.2

Un= uncontrolled from the total sample per study; CON=controlled from the total sample per study

Calculation of Odd ratio by Sample size

The calculated odds ratios (OR) and 95% confidence intervals (CI) for each study:

Author (Year)	Sample	Odds Ratio	95% CI Lower	95% CI Upper
AL-Jahdali et al, 2008	1060	3.15	2.64	3.76
AL-Jahdali et al, 2012	450	0.09	0.07	0.13
Al-Jahdali et al, 2013	450	0.09	0.07	0.13

Al-Jahdali et al. 2019	1009	0.37	0.31	0.45
Al-Zahrani JM et al. 2015	400	0.44	0.33	0.58
BinSaeed, 2015	260	4.55	3.15	6.58
GhalebDailah, 2021	263	0.14	0.09	0.20
Habib et al., 2014	53	1.46	0.68	3.14
Tarrafa H et al. 2018	7306	5.77	5.37	6.19
Tayeb et al., 2017	173	2.90	1.87	4.49
Torchyan et al., 2017	257	4.39	3.04	6.36
Overall odd ration 1.23 (0.98 to 1.54)				
overall unadjusted analysis is 0.11 (0.07 to 6.58)				
