# Supplementary figures and images for: Asthma Control Among Adults in Saudi Arabia: A Systematic Review and Meta-Analysis
Source: J Clin Med. 2025 Aug 14;14(16):5753. doi: 10.3390/jcm14165753 (PMC12386509; doi:10.3390/jcm14165753)

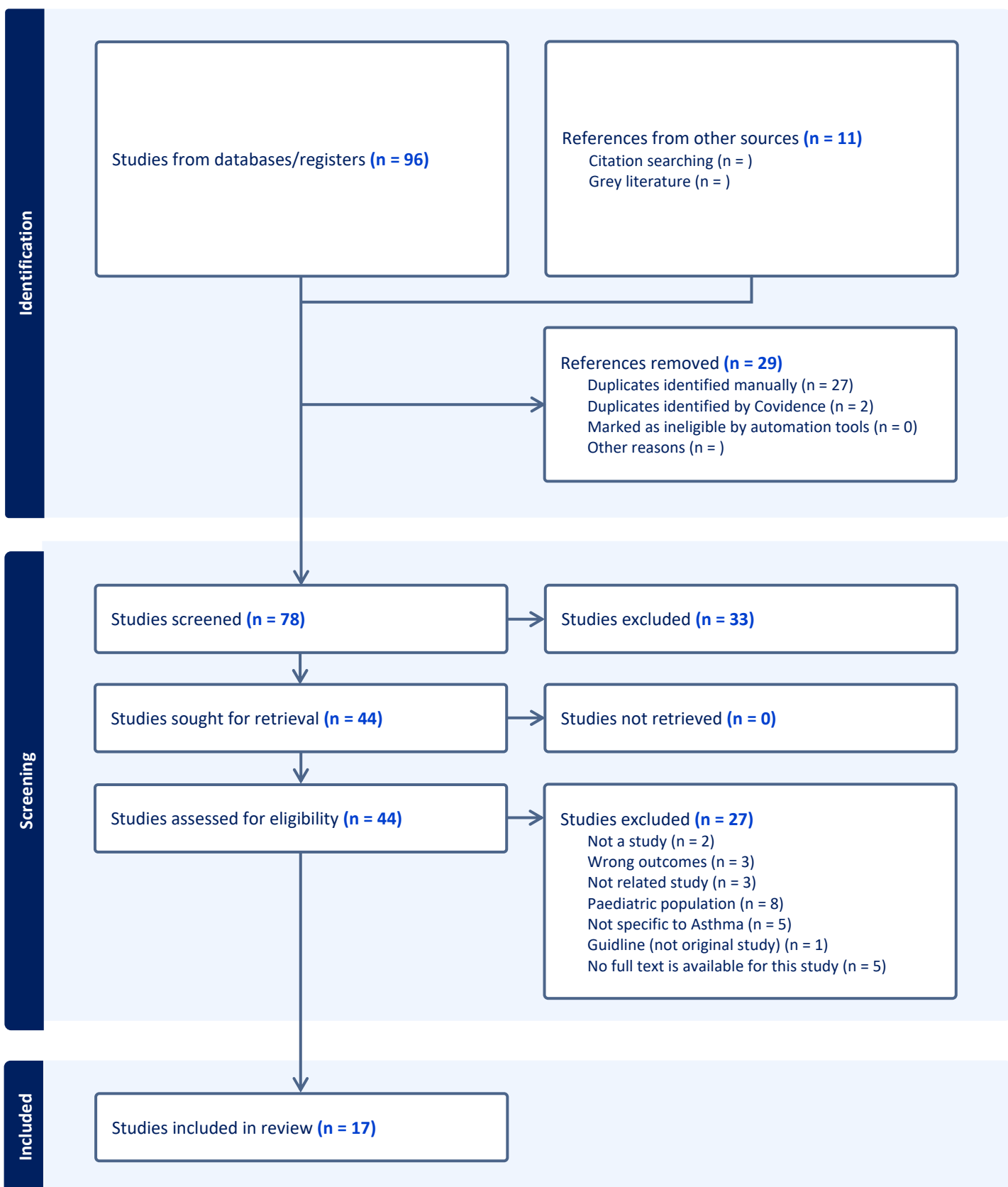

Supplement: Supplementary file 1 [file jcm-14-05753-s001.zip › jcm-3686807-Figure S1.pdf]
